# Phenological diversity in wild and hybrid grapes (*Vitis*) from the USDA-ARS cold-hardy grape collection

**DOI:** 10.1038/s41598-021-03783-x

**Published:** 2021-12-21

**Authors:** Benjamin Gutierrez, Heidi Schwaninger, Victoria Meakem, Jason Londo, Gan-Yuan Zhong

**Affiliations:** 1 United States Department of Agriculture-Agricultural Research Service, Plant Genetics Resources Unit, 630 W. North St., Geneva, NY 14456 USA; 2 United States Department of Agriculture-Agricultural Research Service, Grape Genetic Research Unit, 630 W. North St., Geneva, NY 14456 USA

**Keywords:** Genetics, Plant sciences, Plant breeding

## Abstract

Wild grape relatives and hybrids have been useful in breeding for tolerance to biotic and abiotic stress, however, few studies have emphasized wild and hybrid grapevines for phenological diversity. Utilization of phenological diversity in grapevine breeding could facilitate expansion of grape production into more varied climate regions. Budbreak, bloom, and veraison observations for 1583 accessions from 20 taxa from the United States Department of Agriculture *Vitis* collection in Geneva, New York, USA. Genotypic and species variation were estimated. *Vitis vinifera* ancestry was estimated in *Vitis* hybrids using principal components analysis. Observations ranged 26.6–162.1 (79–141 JD) with an average of 82.6 GDD (118 JD) for budbreak, 206.8–1055.2 (141–222 JD) with an average of 371.9 GDD (163 JD) for bloom, and 849.9–1627.0 (202–290 JD) with an average of 1207.9 GDD (235 JD) for veraison. Seasonal correlations were high for bloom and veraison (0.85–0.95) and moderate for budbreak (0.61–0.65). Moderate heritability was estimated for veraison (0.62) and bloom (0.49), and weak heritability for budbreak (0.2). The species effect was greatest in bloom and explained 42% of the variation, with increasing bloom GDD associated with increasing contribution of *V. vinifera* in *Vitis* hybrids.

## Introduction

Phenology, the study of the relationship between climate and timing of periodic biological phenomena, is of critical importance for agricultural crops in view of anticipated future climatic conditions. Shifting environmental conditions are particularly challenging for long-lived woody perennial species, which can take decades to develop new cultivars and establish commercial plantings. Clonal propagation is used to preserve unique allele combinations of a cultivar which are otherwise altered through sexual reproduction. In grapevine (*Vitis* L.), some of the most popular cultivars originated hundreds^1^ or possibly a thousand years ago^2^, largely unchanged from their progenitors. While cultivars remain fixed through propagation, grape producing regions are predicted to shift world-wide^3–5^. Adapting grapevine cultivars to changing environmental conditions is a major challenge for future production^6^.

Adequate heat, absence of extreme heat and severe frost damage set the most prominent environmental boundaries for sustainable grape production^7^. Cultivated grapevines, including *Vitis vinifera*, *Vitis* interspecific hybrids, and muscadine grapes, are among the most culturally and economically valuable fruit crops worldwide, with production favoring *V. vinifera*. Genetic diversity held in germplasm collections can facilitate development of new cultivars which are better adapted to growing conditions in the current and future grape-growing areas^8,9^.

Diverse genetic resources hold tremendous potential for crop improvement. Wild grapevine genetic resources have been utilized for disease and pest resistance, as well as for cold-hardy breeding^10^. However, introgression of traits from wild germplasm often comes at the expense of fruit quality and requires thorough characterization of genetic resources, planning, and long-term breeding. Cultivars, hybrids, and wild species can have significant phenological differences. Knowing the phenological characteristics of a taxonomically broad suite of *Vitis* germplasm is critical for developing cultivars adapted to current and predicted climates in grape growing regions. Other grapevine phenological studies report significant variation, though focused primarily on *V. vinifera*^11–13^. Conversely, the phenological diversity of wild or hybrid grapevines under common environmental conditions is rarely reported.

Grapevine seasonal development has three major time points: budbreak, when vegetative growth commences after dormancy, bloom, and veraison, the onset of ripening characterized by the initiation of sugar accumulation and rapid pigmentation of the berries in colored varieties^14^. Progress through these three stages is controlled primarily by temperature but other exogenous and endogenous factors contribute, especially during the ripening phase^14,15^. Critical temperatures for directing grapevine development include the chilling requirement during the dormant season and heat units during the growing season. Chill units are thought to accumulate between 0 and 7.2 ℃ for 50–400 h to satisfy endodormancy requirements in *V. vinifera*^16,17^ while other *Vitis* species may require significantly more^18^. Growing Degree Days (GDD) are one commonly used measure of heat accumulation during the growing season.

The objectives of the present study were to (1) phenotype the collection of grapevines held at the repository of the United States Department of Agriculture (USDA) Plant Genetic Resources Unit in Geneva (PGRU), Geneva, New York, USA (approximately 1426 permanent accessions) across multiple years for the phenological traits date of budbreak, bloom, and veraison, (2) classify accessions relative to budbreak, bloom date and veraison, and (3) give access to data for research and cultivar development. Characterization of budbreak, bloom, and veraison of diverse germplasm will increase the usefulness of the collection to facilitate the utilization of these resources for grapevine breeding and research.

## Materials and methods

### Plant material

All evaluated grapevines were maintained by the USDA-ARS PGRU as part of the National Plant Germplasm System and were publicly available for research and breeding. The grape repository consisted of 1596 accessions, including 2 genera (*Vitis* and *Ampelopsis* Michx.), and 20 species and hybrids (Table 1). The vineyard was located at Geneva, New York, USA on the Cornell AgriTech campus at an altitude of 198 m in USDA plant hardiness zone 6a (characterized by average annual extreme minimum temperatures of − 23.3 to − 20.6 ℃). The soil was fertile Ontario Loam. The vines were trained to an Umbrella Kniffin System planted 1.83 m (6 ft) apart within rows and 3.048 m (10 ft) between rows. The vines were managed in accordance with routine commercial practices as to weed and pest control, fertilization, and pruning. Dormant pruning occurred from January to February. The vineyard was not irrigated due to ample natural moisture. Most accessions were planted as two replicated, self-rooted vines planted side-by-side. Additional vines belonged to seedling families. The age of the vines varied due to date of introduction and regeneration of original vines. Taxonomic identification was determined through accession passport records and National Plant Germplasm System curators and collaborators, using genetic markers to make corrections, as highlighted by Klein et al.^19^. Botanical nomenclature is based on GRIN-Global^20^.

**Table 1:** Number of *Ampelopsis* and *Vitis* taxa evaluated from the USDA-ARS PGRU *Vitis* collection in Geneva, NY.

Species	No.	Species	No.
*Ampelopsis cordata* Michx	2	*V. labrusca* L	38
*A. delavayana* Planch	3	*V.* × *novae-angliae* Fernald	1
*A. glandulosa* (Wall.) Momiy	9	*V. palmata* Vahl	3
*V. acerifolia* Raf	66	*V. piasezkii* Maxim	2
*V. aestivalis* Michx	28	*V. riparia* Michx	173
*V. amurensis* Rupr	22	*V. romanetii* Rom. Caill	2
*V.* × *andersonii* Rehder	1	*V. rupestris* Scheele	37
*V.* × *champinii* Planch	2	*Vitis* spp.	50
*V. cinerea* (Engelm.) Millardet	48	*V. vinifera* subsp. *vinifera* L	21
*V. ficifolia* Bunge	1	*V. vulpina* L	40
*Vitis* hybrid	1024		

Nomenclature based on GRIN-Global^20^.

### Phenological stages and scoring

Observations on budbreak, bloom, and veraison were taken throughout the three growing seasons of 2011, 2012, 2013 on all accessions (approx. 2500 vines). Additional budbreak and bloom data for 2008 and 2009 was retrieved from GRIN-Global^20^. Vines were scored weekly until a specified stage on the extended scale of the Biologische Bundesanstalt, Bundessortenamt und Chemische Industrie (BBCH)^21,22^ was reached. The target stage for budbreak was 05, “Wool stage”, where brown wool is clearly visible in 50% of buds, considering only the middle parts of canes, and disregarding spurs and the first and last two buds on each cane^23^. Full bloom was reached at stage 65, when 50% of the flower hoods had fallen. Veraison on female and hermaphroditic vines was reached at stage 83 when 50% of berries developed color. If the target stage was not present on an observation day, it was linearly interpolated from successive scorings. Full maturity was not scored due to the complexity of assaying many highly diverse cultivated and wild accessions.

### Meteorological data

Data was obtained from the Network for Environment and Weather Applications (NEWA) Geneva, NY station located within 3 km of the repository vineyard (https://newa.cornell.edu). In Geneva, achieving the chilling requirement is not a limiting factor and growing degree days (GDD) was considered the temperature component most strongly influencing phenology. Accumulated GDD were determined using the Baskerville-Emin (BE) sine wave algorithm to account for variable spring weather^24^ with a base temperature of 10 ℃ and accumulation beginning on day 60^25^ and ending on day 305.

### Genotyping-by-sequencing

Genotyping-by-sequencing (GBS) data for wild *Vitis* accessions were developed by Klein et al.^19^ and DNA isolation, library construction, and sequencing for *Vitis* hybrids followed these methods. Raw data was merged for both wild and hybrid accessions and SNPs were discovered and called using the TASSEL 5.0 pipeline^26^ aligned to the *V. vinifera* PN40024 12X.v2 reference genome^27,28^ using Burrows–Wheeler Alignment^29^, resulting in 885,630 sites across 1744 individuals. Sites were filtered for minor allele frequency of less than 0.05, more than 0.20 missing sites, and mean depth of 8, and 215 individuals were removed for poor depth. Sites were also filtered for linkage disequilibrium using an *r* threshold of 0.20. Final genotype matrix included 1529 individuals and 19,249 SNPs.

### Data analysis

The data were analyzed using R version 3.5.3^30^. Analyses used the mean of replicate genotypes or seedling families within year or averaged across years. Mosaic plots created with ‘vcd’ package^31^. Variance components for the random effects of genotype, species, and year for each trait were determined using ‘lme4’^32^. Principal Components Analysis (PCA)-based ancestry estimation was used to determine *V. vinifera* ancestry of *Vitis* hybrids^33,34^. PCA was performed using ‘snprelate’^35^ package in R on a set of 14 *V. vinifera* cultivars and 14 North American *Vitis* species *V. acerifolia*, *V. labrusca*, *V. riparia,* and *V. rupestris*, with remaining *Vitis* hybrids projected onto PC1 and PC2 axes. The percentage of *V. vinifera* was determined for *Vitis* hybrids with the formula:

$$\% \;V.\;vinifera = {\text{b}} \div ({\text{a}} + {\text{b}}) \times 100,$$

where ‘a’ and ‘b’ represent the Euclidian distance from PC1 eigenvector values for *Vitis* hybrids and the mean PC1 values for *V. vinifera* and *Vitis* species, respectively.

## Results

### Phenological variation

**Figure 1:**
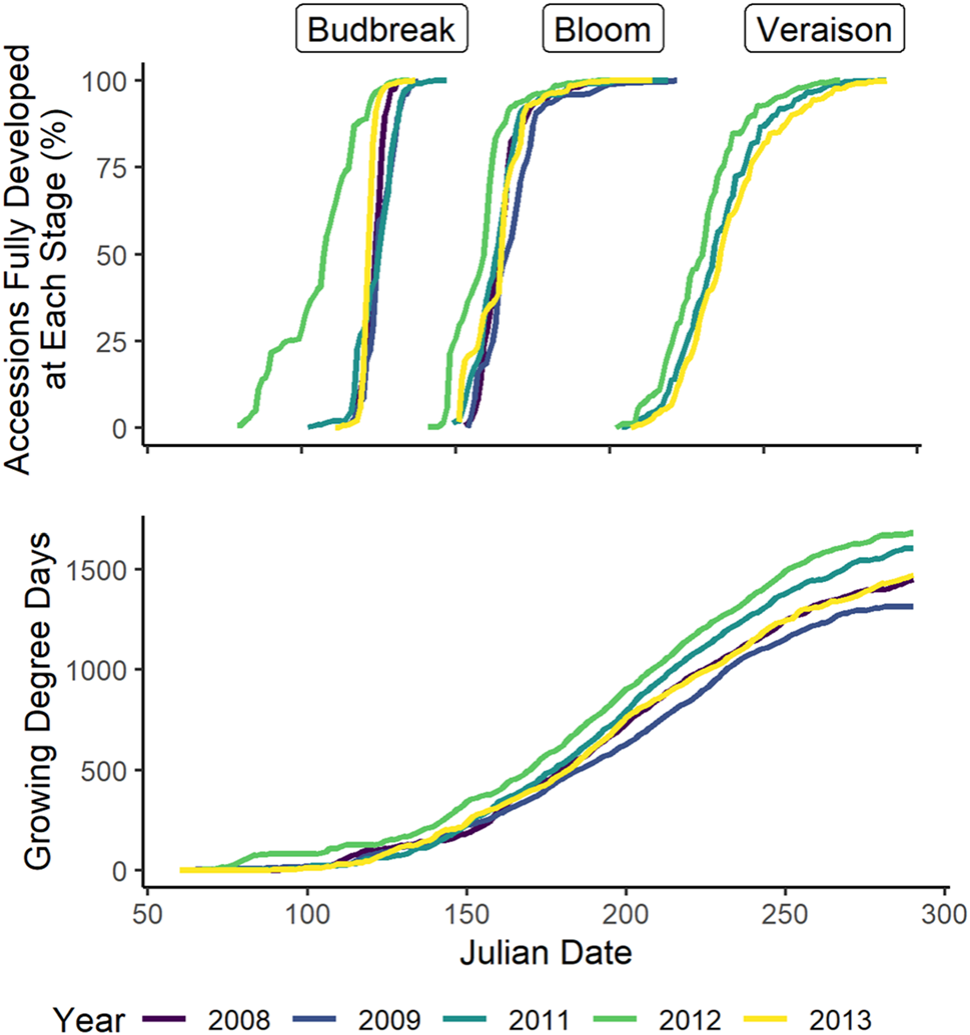
Percentage of accessions that reached budbreak, full bloom and veraison (**A**) and Accumulated Growing Degree Days (base 10 ℃) in Geneva, NY USA (**B**) from day 60 to 305 for the years 2008, 2009, 2011, 2012, and 2013.

Growing degree days for budbreak (BB), bloom (BL), and veraison (V) were determined for 1583 accessions from 20 species (Table 1) across 5 years. Annual accumulated GDD from day 60 to 305 ranged from 1342.7 to 1714.7 GDD (Fig. 1). On average, BB was first observed once GDD reached a value of 47.3, which typically occurred between days 102 and 117. However, in 2012 the accumulation of GDD between days 60 and 100 was more than 12 times higher than average, causing BB to begin nearly 30 days earlier than other years (Fig. 1). In contrast, the onset of BL and V was more consistent across years, beginning between days 141–154 and 202–207, respectively, once GDD reached an average value of 230.9 and 871.0 (Fig. 1).

**Figure 2:**
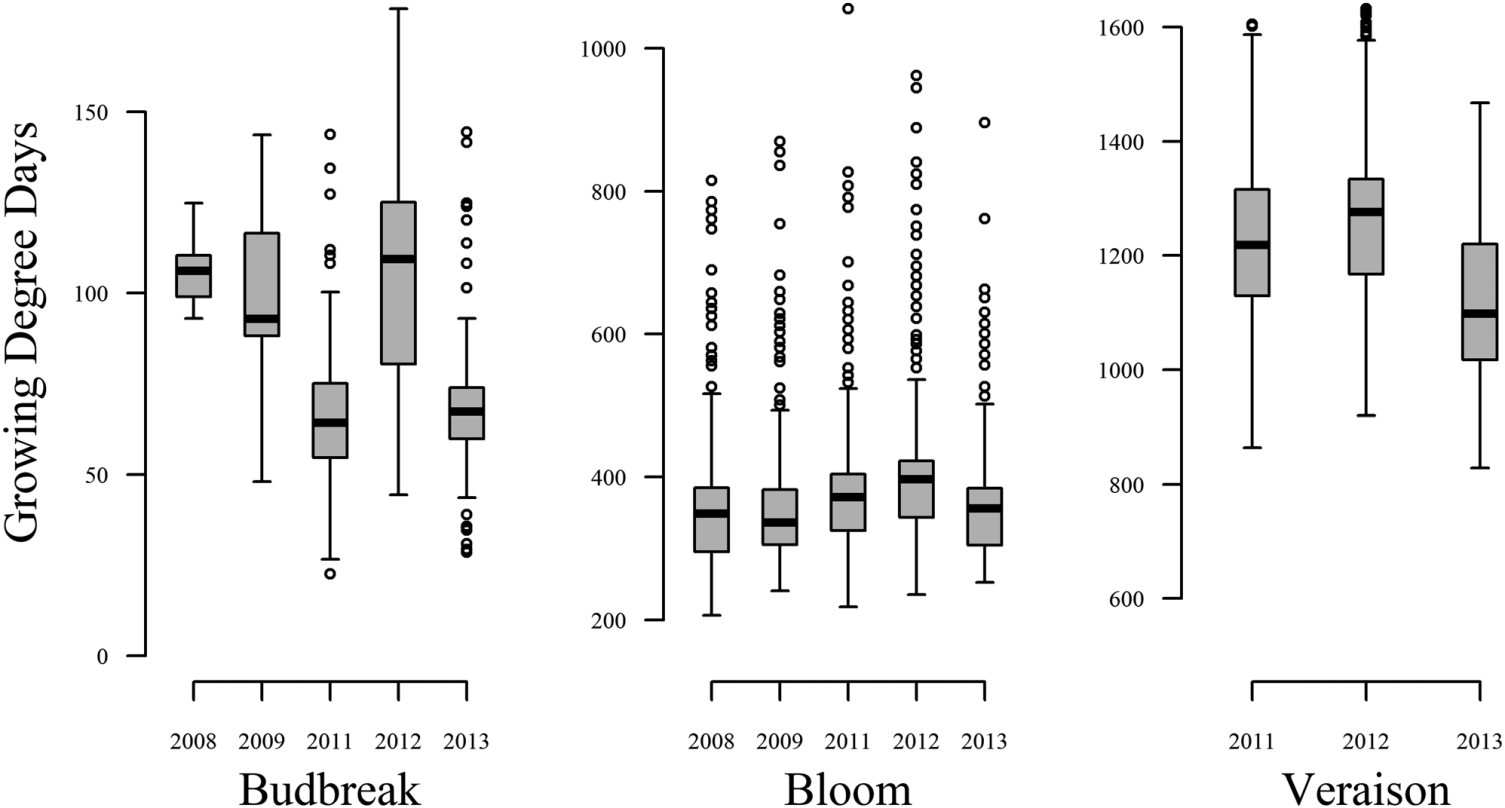
Phenological variation by year in the USDA *Vitis* collection in Geneva, NY, USA.

Phenological variation is depicted in Fig. 2 and summarized in Table 2 including variance components for genotype, species, year effects. There were considerably more outliers associated with BL across all years, with 96 accessions falling outside the expected maximum range (Q3 + 1.5× interquartile range), of which 45 were *V. cinerea*. Among the bloom outliers, 53 accessions were considered outliers for at least three years, with 33 as outliers for all five years. BB was the least stable across years with pairwise correlation coefficients ranging from 0.61 to 0.76 with Year accounting for 55% of the observed variation. For BL and V, correlation coefficients ranged from 0.92 to 0.95 and 0.85–0.89, with Year accounting for 5% and 20%, respectively.

**Table 2:** Summary of variation in the USDA-ARS PGRU *Vitis* collection for budbreak (BB), bloom (BL), veraison (V), and their intervals reported in GDD.

Trait	No. days	GDD Min	GDD Mean ± SD	GDD Max	Seasonal correlations	Genotype	Species	Year	Residual	Heritability
BB	79–141	22.6	82.4 ± 25.4	178.4	0.61–0.76	152.1	66.5	411.7	117.8	0.20
BL	141–222	206.8	371.9 ± 83.7	1055.2	0.92–0.95	4349.9	3729.2	446.8	352.2	0.49
V	202–290	828.9	1207.9 ± 148.8	1631.9	0.85–0.89	15,215.0	2318.0	4777.0	2423.0	0.62
BB_BL	–	115.7	291.2 ± 78.5	1000.6	0.88–0.95	3487.4	2842.5	945.9	360.8	0.45
BB_V	–	782.5	1126.2 ± 134.2	1550.2	0.85–0.89	14,435.0	1885.0	3095.0	2445.0	0.66
BL_V	–	492.7	829.1 ± 115.1	1313.1	0.80–0.84	10,899.0	509.0	2683.0	2485.0	0.66

Variance components for genotype, species, year, and residual error, with heritability = genotype/sum of variance components.

**Figure 3:**
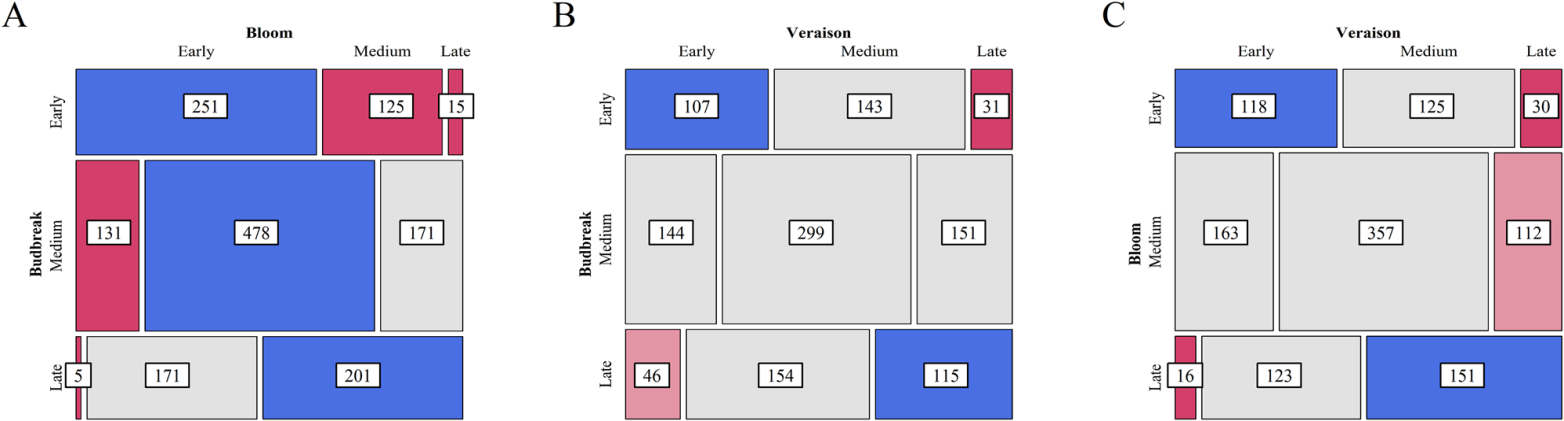
Mosaic plot of phenological class (Early, Intermediate, and Late) for (**A**) budbreak and bloom, (**B**) budbreak and veraison, and (**C**) bloom and veraison. Number of accessions within each group is included. Color and shade denote deviation from expected observations, with greater (blue) or fewer (red) than expected proportions or no significant difference (gray) assuming independence.

There were low to moderate correlations between traits, with Pearson correlation coefficients of 0.29, 0.53, and 0.57 for BB/V, BL/V, and BB/BL pairs, respectively. Accessions were classified for each phenological state as ‘Early’ (< 25th percentile), ‘Late’ (> 75th percentile), or ‘Intermediate’ if within the 25th and 75th percentiles based on mean GDD across years. An independence test of trait pairs showed a strong association among like classes (Fig. 3). For example, early budbreak was strongly associated with early bloom and veraison. Contrasting budbreak and bloom, there were five accessions with late budbreak and early bloom including three *V. rupestris*, one *V. riparia*, and one *Vitis* hybrid accessions. There were 46 accessions with late budbreak and early veraison, including 40 *Vitis* hybrids, 5 *V. rupestris*, and *Vitis vinifera* ‘Zefir’.

### Species and accession variation

**Figure 4:**
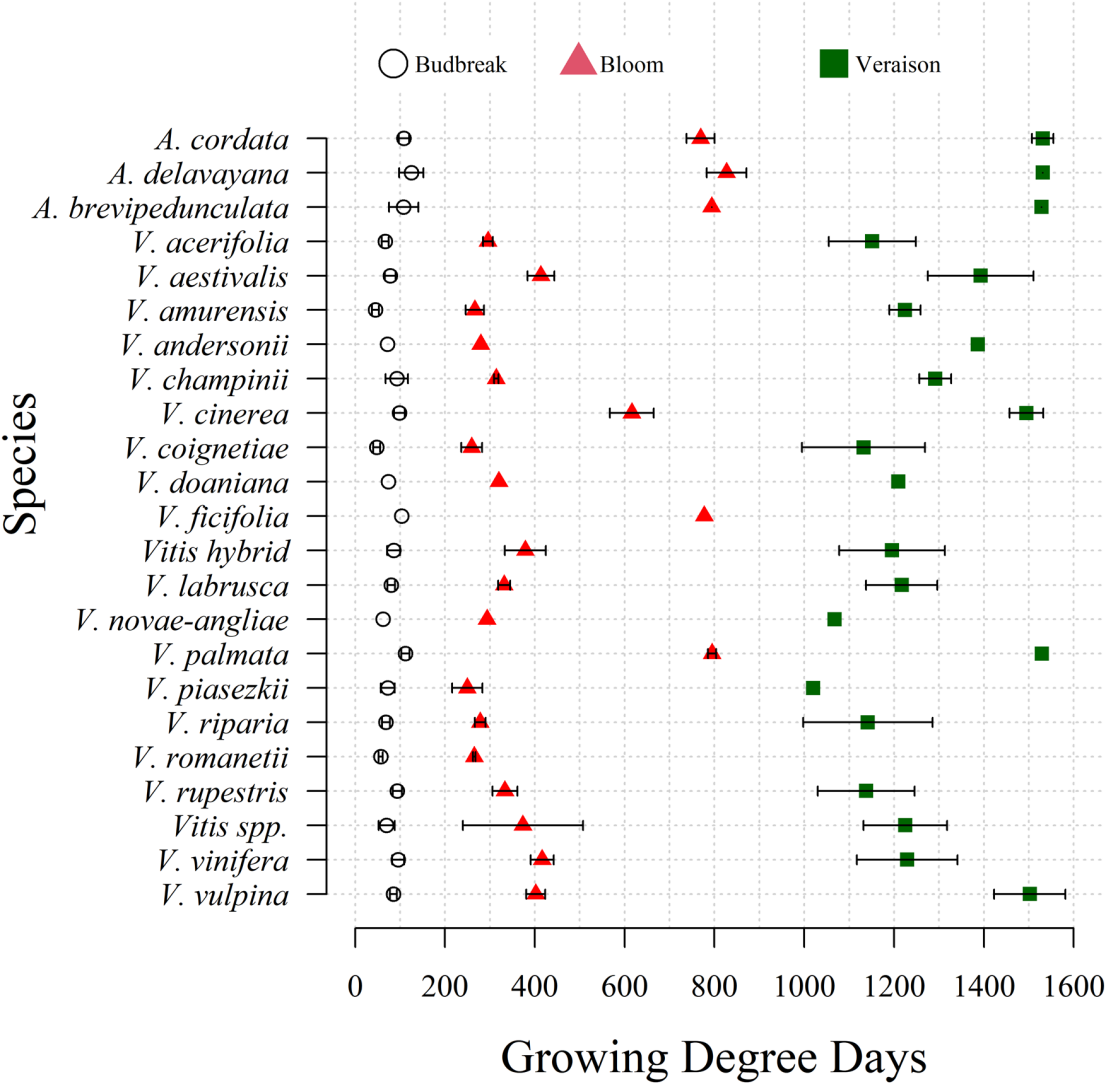
Variation for phenological stages budbreak (circle), bloom (triangle), and veraison (square) in the USDA-ARS PGRU *Vitis* collection by species. Error bars depict variation in taxa with > 1 accession for each stage.

Heritability and species effect varied with each trait (Table 2). Heritability was higher for V (*H*^2^ = 0.62) and BL (*H*^2^ = 0.49) than for BB (*H*^2^ = 0.20). There was significant phenological variation across species (Fig. 4). BL was impacted the most by species variation (42% of the total variance explained), whereas species explained < 10% of the BB and V variation. Within species variation was minimal for BB. For BL, accessions of *Ampelopsis* spp. (n = 14), *V. cinerea* (n = 48), *V. ficifolia* (n = 1), and *V. palmata* (n = 3), required substantially more GDD to achieve bloom and veraison, with *V. ficifolia* never reaching full veraison in Geneva, NY, USA. The greatest within species variation for BL was observed in *V. cinerea* (n = 48, 394.8–738.3 GDD) and *Vitis* hybrids (n = 1024, 256.3–628.6 GDD), excluding unclassified accessions listed as *Vitis* spp. (n = 50, 273.2–1055.2 GDD). Considering the interval from BB to V, *V. rupestris* (n = 37) and *V. riparia* (n = 173) require the least GDD to reach all three stages, with 1044.1 and 1072.7 GDD, respectively, and *V. vulpina* (n = 40) and *V. palmata* (n = 3) the most, with 1417.0 and 1,417.5 GDD, respectively. Among the taxa evaluated, *V. acerifolia* (n = 6) and *V. riparia* (n = 30) had several accessions classified as Early in all three stages, and *V. cinerea* (n = 21) and *A. glandulosa* (n = 7) with accessions classified as Late in all three stages.

**Figure 5:**
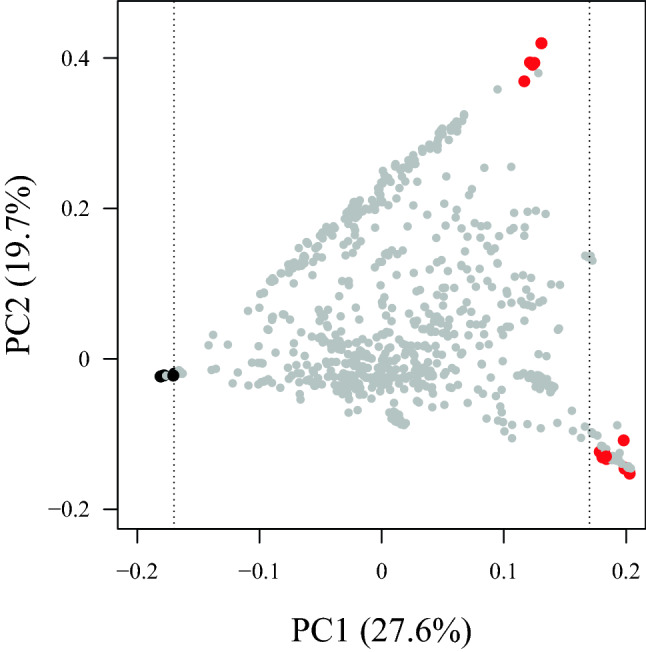
Principal Component Analysis of *Vitis* vinifera (n = 14, black), wild *Vitis* species (n = 14, red), and projected *Vitis* hybrids (n = 721, gray) based on 19,226 SNPs. Axes show PCs 1 and 2, capturing 47.3% of the variation. Dotted vertical lines represent mean PC1 values for *V. vinifera* and wild *Vitis* genotypes.

**Figure 6:**
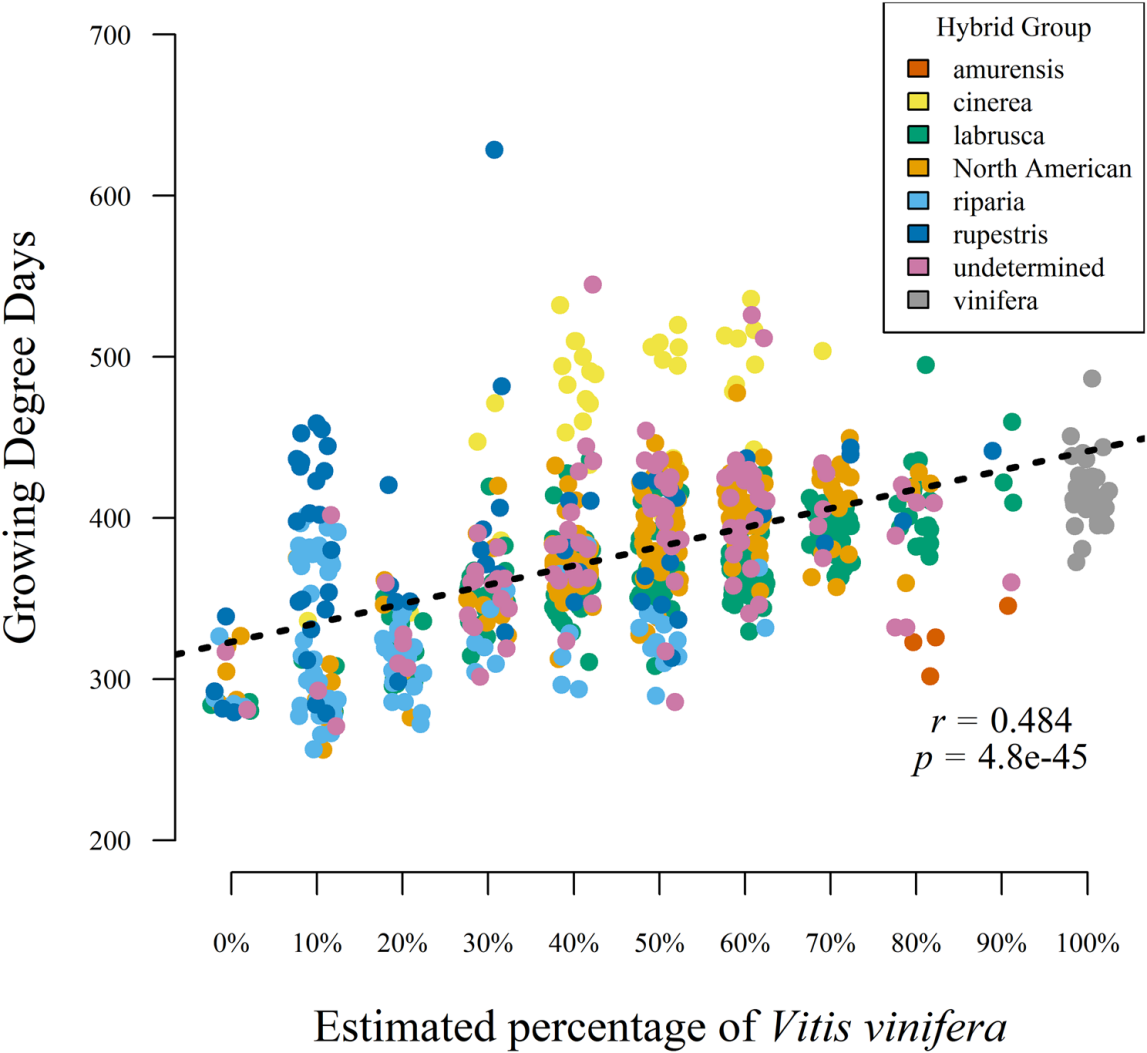
Bloom phenological variation of 721 *Vitis* hybrids grouped by estimated percentage of *Vitis* vinifera. Model R2 and p-value included with regression line in black. Color indicates the primary wild progenitor in the hybrid. North American (orange) group represents hybrids with two or more North American *Vitis* species. Undetermined (pink) accessions are hybrids with no pedigree information.

PCA-based ancestry estimates were used to determine the relationship of 721 *Vitis* hybrids to *V. vinifera* to better evaluate the phenological diversity of this group (Fig. 5). PCs 1 and 2 captured 27.6% and 19.7% of the variation of 14 *V. vinifera and* North American wild *Vitis.* PC1 separates *V. vinifera* (black) from wild *Vitis* (red), and PC2 separates *V. labrusca* (top right) from the other wild *Vitis* species. Accessions classified as *Vitis* hybrids were projected into this space. There is some tight clustering towards the center of the plot, and the *V. labrusca* hybrids, such as ‘Concord’, are plotted diagonally between the *V. vinifera* and *V. labrusca* clusters. *V. vinifera* ancestry was estimated using PC1 values, and were consistent with previous reports^33,34^ and known pedigrees. Percentage of *V. vinifera* ranged from ~ 0.0 to 99.3%, with an average of 44.8 ± 21.21%. Among the hybrids, 310 of 721 had > 50% *V. vinifera* ancestry and 411 had < 50% *V. vinifera* ancestry*.* Several of accessions listed as hybrids had ancestry estimates of > 95% *V. vinifera* and were hereafter considered to be full *V. vinifera*. The effect of species is highlighted by the significant association of bloom date with increasing percent of *V. vinifera* with an *R*^2^ = 0.247 (Fig. 6). Additional pedigree information for wild progenitors in hybrids was retrieved from GRIN-Global^20^ and the *Vitis* International Variety Catalogue^36^. Most hybrids in the USDA collection originated from North American species. Color groupings in Fig. 6 show additional separation of hybrid accessions, particularly for *V. cinerea* (yellow) hybrids with higher bloom GDD and *V. riparia* (light blue) hybrids with lower bloom GDD.

## Discussion

Budbreak, bloom date and veraison are critical milestones for grape production and key characteristics in the breeding of climate adapted cultivars. Grape production favors the European wine grape (*V. vinifera*) adapted to Mediterranean climates. However, modern grape production increasingly occurs in climates less suited for *V. vinifera*, with low winter temperatures, late spring frosts, and short growing seasons. A broad base of genetic diversity, including variation of phenological traits, is required to develop climate adapted grape cultivars with an aim of sustainable production. We observed broad phenological variation that could be utilized for climate adaptation. Budbreak in the PGRU *Vitis* collection occurs within a narrow window across species and accessions with low heritability (H^2^ = 0.20). The low heritability yet moderate seasonal correlations (0.65–0.76) for budbreak in this study may result from inadequate modeling using GDD without considering chilling requirements and winter de-acclimation associated with this trait. Other possibilities may include differential response between accessions and vineyard management, including timing of pruning^37^.

Veraison and bloom were better modeled by GDD, with higher heritability (0.62 and 0.49, respectively) and reduced seasonal variability. Species effect was high for bloom, accounting for 42% of the observed variation. Conversely, Species accounted for < 10% of the variation in budbreak and veraison. Veraison was spread across more days from the earliest to the latest accession than either budbreak or flowering time with the latter occurring most concentrated. The same pattern was observed in other studies that measured veraison in units of heat, and has been ascribed to heat summation having larger absolute values in late developing cultivars, and to other processes like crop management, water status or clonal variability^11,12,38^.

Hybrids represent the largest group in the Geneva repository. *Vitis* hybrids are valued for their cold-hardiness, disease tolerance, and unique fruit quality, particularly the aromatic qualities of *V. vinifera* × *V. labrusca* hybrids. However, unlike other taxonomic assignments, the broad assignment to ‘hybrid’ fails to capture the unique composition of this group. An estimate of the genetic composition of hybrids helps to access the broad genetic variation contained within this group. Contribution of *V. vinifera* depends on improvement status and number of backcrosses using *V. vinifera.* For example, we estimated ‘Concord’ and ‘Niagara are 36.9 and 50.2% *V. vinifera,* respectively. The average of 44.8*% V. vinifera* ancestry suggests that many of our hybrids are first generation interspecific crosses, with more accessions (411/721) having less than 50% *V. vinifera* ancestry. The utility of grouping *Vitis* hybrids by percentage of *V. vinifera* ancestry was highlighted by the pattern of increasing bloom GDD with increasing percentage of *V. vinifera.*

The large phenological diversity revealed in this study has many potential uses. Existing cultivars could be established in adapted climates, or these resources can be used to develop new cultivars based on phenological traits and other qualities. Genetic traits are being analyzed and markers developed for marker-assisted breeding^6,39^. Phenotypic variation among cultivars suggests a genetic component to phenological tendencies. This is further noted in many pedigreed crosses of early blooming with late blooming genotypes tend to produce offspring with intermediate phenotype. For example, the hybrid Millardet 420 (accession number PI 279058), an important rootstock cultivar, is a cross of *V. cinerea* (late bloom) × *V. riparia* (early bloom) and their hybrid offspring blooms intermediately. There appears to be a significant association between timing of developmental stages for each trait. For example, accessions with early budbreak also tend to bloom early as well, although we identified several accessions with contrasting classifications, such as 46 accessions with late budbreak and early veraison. Late budbreak cultivars will help to reduce frost damages such as are observed in the American Midwest and Northeast during vine de-acclimation. Late budbreak and early ripening cultivars will support the expansion of viticulture into regions with short growing seasons where *V. vinifera* cultivars are poorly adapted.

Because this study was conducted in one location in a common garden experimental design, each genotype represented by a small number of vines, full maturity was never assayed and cultivar or genotype characteristics cannot easily be extrapolated because grapevines are sensitive to local environmental factors contributing to terroir^40–42^. However, accessions can be compared to each other, allowing the informed and efficient selection of germplasm for further use, and thus fulfilling the mayor goal this study; to facilitate the specific, researcher-driven use of the germplasm collection. Phenological data for all accessions ancestry estimates for *Vitis* hybrids are provided through GRIN-Global^20^.

## Conclusions

Understanding phenological variation regarding heritability, species variation, and seasonal stability in grapevine genetic resources will help promote their utilization for research and targeted breeding of adapted cultivars. Moderate heritability in veraison and bloom are promising for breeding programs, particularly with genetic resources with additional traits of interest. Physiological requirements for budbreak are also controlled by chilling hours which were not evaluated in this study and could explain the low heritability when modeled by GDD alone. A phenology data base has been developed that includes Julian Day and heat units at budbreak, bloom time and veraison of a diverse collection of 1583 cold-hardy grape genotypes including 788 hybrid grapes and 13 wild species. To maximize the usefulness of the data base, access is provided through supplementary data files to the yearly and summary data of all accessions. These data are intended to be used by grape breeders and researchers aiming to develop cultivars adapted to novel or altered grape growing regions. To aid in the selection of accessions, additional accession-specific information such as fruit, leaf and flower images and values of a range of descriptors can be accessed through GRIN-Global^20^.

## Data Availability

Phenological and ancestry estimates data are available through the Genetic Resources Information Network (GRIN-Global) under the GRAPE Crop descriptors (https://npgsweb.ars-grin.gov/gringlobal/crop?id=174).
